# A novel missense mutation in the FERM domain containing 7 (*FRMD7*) gene causing X-linked idiopathic congenital nystagmus in a Chinese family

**Published:** 2013-08-06

**Authors:** Zhirong Liu, Shanying Mao, Jiali Pu, Yao Ding, Baorong Zhang, Meiping Ding

**Affiliations:** Department of Neurology, the Second Affiliated Hospital of Zhejiang University School of Medicine, Hangzhou, China

## Abstract

Purpose: Idiopathic congenital nystagmus (ICN) is a genetically heterogeneous disease. Thus far, the disease gene has been identified as the FERM domain containing 7 (*FRMD7*) gene. The purpose of this study was to elucidate the clinical and genetic characteristics of a four- generation Chinese family with ICN.

Methods: The clinical data and the genomic DNA of a Chinese ICN family were collected following the provision of informed consent. All coding exons of the *FRMD7* gene were amplified by PCR and then sequenced. Afﬁnity GST-p21 activated kinase 2 (PAK2) precipitation was used to investigate whether this novel *FRMD7* mutant influenced Rac1 signaling activation in the human embryonic kidney 293 T cells (HEK 293T) cells transiently cotransfected with wild-type or mutant FRMD7 and Rac1.

Results: A novel missense mutation (c.635T>C) was identified in all affected members. Obligate female carriers were heterozygous in these mutations and the affected males were homozygous, consistent with X-linked inheritance. This mutation is a substitution of proline for leucine. Function analysis showed that this novel mutant influences Rac1 signaling in human HEK 293T cells.

Conclusions: This study widens the mutation spectrum of the *FRMD7* gene. This mutant was shown to activate GTPase Rac1 signaling in vitro; however, the quantity of activated Rac1 was obviously decreased compared with the wild type (p<0.05). Taken together, our data strongly support the hypothesis that the identified *FRMD7* mutant influences GTPase Rac1 signaling, which regulates neurite development. This mutation may be related to the pathogenesis of X-linked ICN.

## Introduction

Idiopathic congenital nystagmus (ICN, OMIM #157640) is an infant-onset disease with the typical features of bilateral ocular oscillations, visual impairment, and abnormal head movement. It has also been termed *congenital motor nystagmus* and exhibits various patterns of inheritance, although X-linked (XL) inheritance with incomplete penetrance and variable expressivity is probably the most common pattern [1,2]. Recent molecular genetic studies have demonstrated that mutations in the FERM domain containing 7 (*FRMD7*) gene are a main cause of XL-ICN. More than 45 different mutations have been reported [3-7]. *FRMD7* contains a conserved N-terminal FERM domain and a FERM-adjacent (FA) domain. FERM domains are characteristic of the band 4.1 superfamily and take their name from the 4.1 (four point one) and ezrin, radixin, and moesin (ERM) proteins. *FRMD7* has been shown to regulate neuronal outgrowth by influencing the dynamics of F-actin during retinoic acid–induced differentiation in mouse neuroblastoma (Neuro-2a) cells [8]. However, the precise mechanism by which this occurs is not clear.

Here, we describe a Chinese family with XL-ICN in whom we have identified a novel mutation of the *FRMD7* gene. Furthermore, we demonstrate that this mutant *FRMD7* influences GTPase Rac1 signaling, which is known to regulate neurite development.

## Methods

### Clinical evaluation and DNA specimens

**Figure 1:**
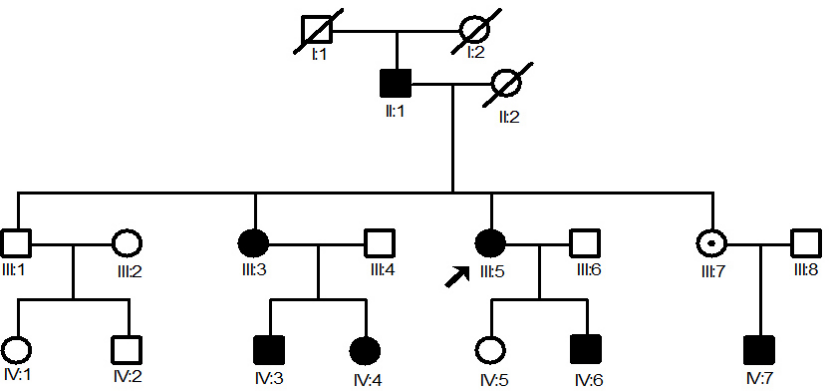
Pedigree of the Chinese family with ICN. The squares and the circles represent males and females, respectively. The index patient is marked with an arrow. The black-filled symbols indicate patients with idiopathic congenital nystagmus, the dotted circles represent female carriers, and a diagonal line symbol indicates a deceased family member.

A four-generation Chinese family with ICN was identified through the Department of Neurology, Second Affiliated Hospital of the Zhejiang University School of Medicine. Informed consent was obtained from all participants in accordance with Zhejiang Institutional Review Board approval. Sixteen individuals participated in the study, including seven affected individuals and nine unaffected individuals (Figure 1).The proband and available family members were evaluated based on a history of neurological examinations. A cranial computed tomography scan was performed in the proband. All the available family members underwent fundoscopic and refractive error examinations. Fundus photographs were recorded by a TRC.50EX Retinal Camera (Topcon Corp. Tokyo, Japan). Blood specimens (5 ml) were collected in EDTA and genomic DNA was extracted by Phenol/chloroform extract from the blood specimens of the sixteen participants.

### Direct sequencing and mutation analysis

The *FRMD7* gene was amplified by PCR using previously published primers [5]. Direct sequencing of the amplified fragments was performed on an ABI Prism 3130 sequencer Genetic Analyzer (Applied Biosystems, Foster City, CA). Sequencing results were assembled and analyzed using the SeqMan II program of the Laser gene package (DNA STAR Inc., Madison, WI). For all samples containing an abnormal *FRMD7* amplicon, new PCR products were reamplified from genomic DNA using the same protocols. Cosegregation analysis was performed.

### Plasmid construction

Full-length *FRMD7* was amplified from previously constructed plasmids [9]. The identity of the PCR product was confirmed by subcloning into the pGEM-T Easy vector (Promega, Madison, WI) and sequencing. Full-length *FRMD7* cDNA was C-terminally FLAG-tagged, digested with BamHI and *Xho*I, and subcloned into pcDNA3.1(+) vector (Invitrogen, Carlsbad, CA). Mutant *FRMD7* (c.635T>C) was constructed by overlap PCR. HA-tagged Rac1 was subcloned into pcDNA3.1(+) vector digested with BamHI and *Xho*I. For prokaryotic expression, the sequence encoding the wild-type (WT) Rac1/Cdc42-binding domain of human p21 activated kinase 2 (PAK2; aa 66–147) was amplified by PCR. The PCR product was confirmed by subcloning into the pGEM-T Easy vector (Promega, Madison, WI) and sequencing. The PAK2-pGEM-T was digested with BamHI and SalI and subcloned into PGEX-5X-1 for expression of glutathione S-transferase (GST) fusion proteins as previously described [10].

### Cell cultures and transient transfections

The HEK 293T cell line was purchased from the Chinese Academy of Sciences Committee Type Culture Collection Cell Bank/Shanghai Institutes for Biologic Sciences Cell Resource Center (Shanghai, China). HEK 293T cells were cultured in Dulbecco’s modified Eagle’s medium (Invitrogen) containing 10% fetal bovine serum (Invitrogen) and 1% penicillin and 1% streptomycin. Cultures were maintained in 5% CO_2_ at 37 °C, and were passaged every two days. Transient transfections were performed using Attractene Transfection Reagent according to the manufacturer’s protocol (Qiagen, Valencia, CA).

### GTPase Rac1 pull-down assay

Bacterially expressed recombinant PAK2 protein was puriﬁed as described previously [11]. *Escherichia coli* strain BL21 (DE3) transformed with the plasmids was incubated for 4 h at 37 °C with 1 mM isopropyl-thio-D-galactoside to induce the expression of proteins which was puriﬁed with a glutathione-Sepharose 4B column. In vivo GTPase Rac1 activation assays were performed according to the protocol of the ProFound Pull-Down GST Protein:Protein Interaction Kit (Thermo number 21,516). HA-tagged Rac1 was cotransfected into HEK 293T cells with FLAG-tagged WT or mutant *FRMD7* using Attractene Transfection Reagent (Qiagen), cultured for 48 h, and lysed. Cell lysates were clariﬁed by centrifugation, and the supernatant was incubated with 100 µg of GST-PAK2 protein immobilized on glutathione-Sepharose beads for 3 min. Beads were washed and eluted in 1X loading buffer. Total protein were separated by sodium dodecyl sulfate polyacrylamide gel electrophoresis (8% gels) and transferred to a PVDF membrane (Bio-Rad, Hercules, CA). After blocking, membranes were incubated with the primary mouse antiflag antibody (Sigma–Aldrich, St. Louis, MO) at 1:4,000 dilution, anti-HA monoclonal antibody (Abmart, Shanghai, China) at 1:2,000, and the membrane-bound antibody was visualized with horseradish peroxidase–conjugated secondary antibody (Abmart, Shanghai, China), diluted 1:5,000. The membranes were processed using the ECL advance western blotting detection kit (Qiagen).

### Statistical analysis

All values are expressed as the mean±standard error of the mean. The differences between the two groups were compared using unpaired *t* tests. A difference of p<0.05 was considered significant.

## Results

### Clinical evaluation

The family pedigree is shown in Figure 1. The disease was clearly transmitted via female carriers to affected males. No male-to-male transmission was identified, indicating that the disease is inherited in an XL dominant pattern. The penetrance of nystagmus within this family varied considerably in the female carriers, but was consistently complete in the male offspring. All seven affected individuals developed nystagmus before 6 months of age and had reduced visual acuity. Normal color vision and fundoscopic examination were recorded in affected individuals. The proband (III:5 in Figure 1), a 39-year-old woman with a five-year history of hypertension and diabetes, was admitted to the Department of Neurology with headache and sudden-onset weakness in her right limbs. Neurological examination on admission showed normal consciousness with a Glasgow coma scale score of 15/15. Her muscle strength was 3/5 in the right upper limb and 2/5 in the right lower limb. She demonstrated a right-sided hemiplegia; the biceps, triceps, and patellar tendon reflexes were all grade 3/4 bilaterally. Hemisensory deficit was found and the right Babinski sign was positive. The proband’s pupils were 3 mm in diameter bilaterally with normal light reflexes. There was conjugate and horizontal nystagmus in both eyes. A cranial computed tomography scan showed a hyperdense lesion in the left putamina that was consistent with cerebral hemorrhage. Chest radiography showed normal results. The laboratory results were as follows: glucose, 198.7 mg/dl (normal: 70 to 110 mg/dl); HbA1c, 10.5% (normal: 4.3%–6.3%); serum sodium, 136 mmol/l (normal: 135 to 145 mmol/l); and serum potassium, 4.33 mmol/l (normal: 3.5–5.5 mmol/l). Urinalysis revealed glucosuria (3+) and ketonuria (-).The clinical features of all participating individuals are shown in Table 1.

**Table 1:** Clinical information on the family with ICN.

Individual	Gender	Age/onset-age	Visual acuity (right/left) and refractive error examination	nystagmus	Abnormal head movement	Neurologic examination	mutation
II:1	Male	72/ 5 month	0.03/0.1 myopia	Conjugate, horizontal	yes	Normal	Hemizygous
III:1	Male	42	1.0/1.2	NO	NO	Normal	NO
III:2	Female	40	1.0/1.0	NO	NO	Normal	NO
III:3	Female	40/4 month	0.2/0. Three myopia astigmatism (right)	Conjugate, horizontal	yes	Normal	heterozygous
III:4	Male	42	1.0/1.0	NO	NO	Normal	NO
III:5(proband)	Female	39/5 month	0.1/0.3 myopia	Conjugate, horizontal	yes	hemiplegia, right Babinski sign was positive	heterozygous
III:6	Male	39	0.9/0.9	NO	NO	Normal	NO
III:7	Female	34	0.8/0.8	NO	NO	Normal	heterozygous
III:8	Male	36	1.0/1.0	NO	NO	Normal	NO
IV:1	Female	15	1.0/1.0	NO	NO	Normal	NO
IV:2	Male	9	1.0/1.0	NO	NO	Normal	NO
IV:3	Male	21/3 month	0.3/0.2 myopia	Conjugate, horizontal	yes	Normal	Hemizygous
IV:4	Female	12/3 month	0.3/0.3 myopia	Conjugate, horizontal	yes	Normal	heterozygous
IV:5	Female	17	1.0/1.0	NO	NO	Normal	NO
IV:6	Male	13/4 month	0.3/0.1 Hyperopia	Conjugate, horizontal	yes	Normal	Hemizygous
IV:7	Male	12/3 month	0.2/0.3 myopia	Conjugate, horizontal	yes	Normal	Hemizygous

This table described the clinical information on affected and unaffected individuals in this family.

### Mutation analysis

**Figure 2:**
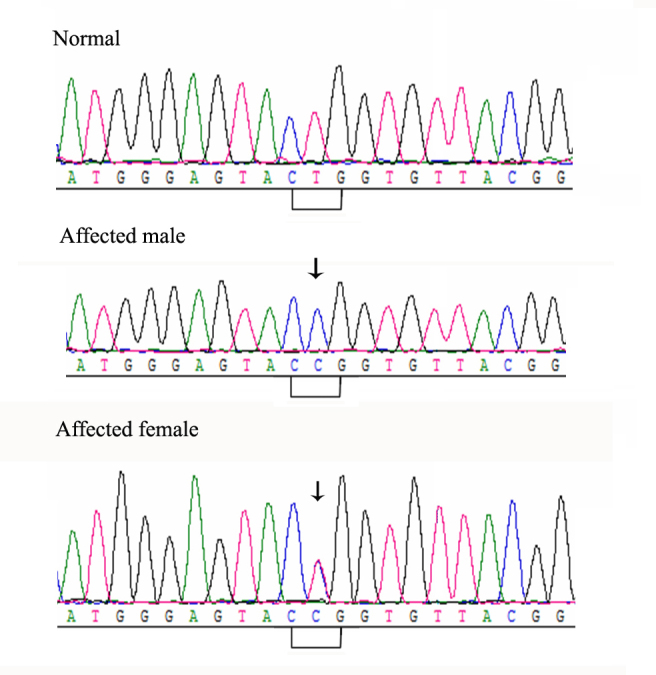
DNA sequence chromatograms of the *FRMD7* gene for affected and unaffected family members. The affected and unaffected family members have been shown in Table 1. Affected family members refers to II:1, III:3, III:5, IV:3, IV:4, IV:6 and IV:7. Unaffected family members refers to III:1, III:2, III:4, III:6, III:7, III:8, IV:1, IV:2, and IV:5.

A novel missense mutation (c.635T>C, in exon 4) of the *FRMD7* gene was identified in all affected members. Obligate female carriers were heterozygous in these mutations and the affected males were homozygous, consistent with XL- inheritance (Figure 2). This mutation, which has not been reported previously, cosegregated with all affected members in this Chinese family, but was not detected in 100 unrelated normal controls or in unaffected pedigree members.

### Novel FRMD7 mutant c.635T>C influences the activation of Rac1 signaling

**Figure 3:**
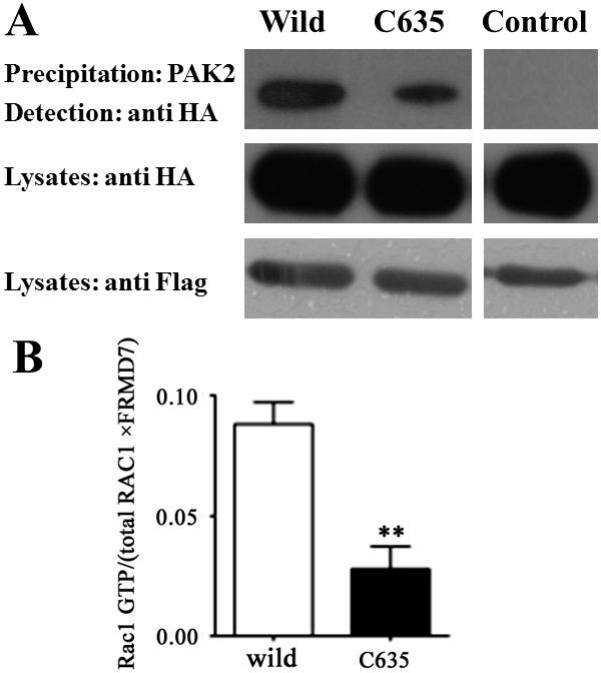
Novel mutation of FRMD7 downregulates the activation of Rac1 signaling HA-tagged human Rac1 was co-transfected into HEK293T cells with Flag-tagged wild-type (Wild) or mutant-type (c.635T>C) FRMD7. The supernatant of cell lysates was incubated with GST-PAK2 protein immobilized on glutathione-sepharose beads, where bound GTP-Rac1 proteins were detected by Western blotting using anti-HA monoclonal antibody. The amount of input HA-Rac1 and Flag-FRMD7 detected by anti-HA or anti-Flag monoclonal antibody. Extracts of HEK293T cells transfected with wild-type FRMD7 could detect the PAK2 precipitation GTP-Rac1 band, however mutant-type FRMD7 contained decreased amounts of GTP-Rac1 compared with the wild-type (**A**). (Wild: wild-type FRMD7+Rac1; C635: mutant-type (c.635T>C) FRMD7+Rac1; Control: empty vector+Rac1). The experiments were repeated five times, and the graphs represent the average of five independent experiments (**B**) (Columns, mean; bars, SEM; *p<0.05).

To investigate whether this novel missense mutation of *FRMD7* influenced Rac1 signaling activation, we used afﬁnity GST-PAK2 precipitation to measure the amount of activated Rac1 in human HEK 293T cells transiently cotransfected with WT or mutant *FRMD7* and Rac1 [10]. The quantity of activated Rac1 induced by this novel missense mutant *FRMD7* (c.635T>C) protein was obviously decreased compared with WT (p<0.05; Figure 3).

## Discussion

We reported a novel heterozygous missense mutation (c.635T>C in exon 4) of the *FRMD7* gene in a family with ICN. The mutant results in an amino acid exchange from leucine to proline, which is a conserved residue and close to the FERM domains that play important role in the function of the *FRMD7* [12]. This mutation in *FRMD7* influences the activation of Rac1 signaling, which might be a potential underlying mechanism for the pathogenesis of XL-ICN. To date, more than 45 different mutations within *FRMD7* have been reported in ICN patients, approximately 75% of which are unique and have only been identified in one ICN family. These mutations are concentrated mainly within the FERM and FA domains, suggesting that these regions play important roles in the function of *FRMD7* [3-6,13-18]. The FERM domain of *FRMD7* is located between amino acids 2 and 282 (ensemble, ENSP00000298542), while the FA domain is located between amino acids 288 and 336 (ensemble, ENSP00000298542). FERM domains have three-lobed “cloverleaf” structures, each lobe representing a compactly folded structure. The FA region is found next to FERM domains in a subset of FERM-containing proteins, suggesting that *FRMD7* is involved in signal transduction between the plasma membrane and cytoskeleton [7,8]. The *FRMD7* gene is also homologous to FARP1 and FARP2, particularly at the N-terminus.

Previous studies have shown that FARP1 and FARP2 are involved in neurite outgrowth and branching [19,20], and it has been recently confirmed that *FRMD7* has a positive effect on this process [21]. On the other hand, more than half of the mutations identified within *FRMD7* are missense. These mutants had a common effect on reducing the neurite length with a varied amount of inhibition for each mutant. *FRMD7* function can be disrupted by destabilizing the protein, disrupting its binding with interacting partners, and/or preventing regulatory modifications to the protein, such as the interaction between *FRMD7* with calcium/calmodulin-dependent serine protein kinase (CASK) during neuronal functioning [7,21,22].

In previous studies, the *FRMD7* protein was shown to be expressed at the actin-rich distal ends of growth cones, affecting the elongation of neurites and therefore suggesting that it may regulate growth cone guidance [8,21]. Rho GTPases are key regulators of actin cytoskeleton dynamics [23]. Therefore, the recruitment and activation of the Rho family of small GTPases (Rac1, Cdc42, and RhoA) and their regulators, which are thought to be the most crucial steps in the formation and movement of the neuronal growth cone, require further investigation [24]. The FERM domain containing the protein radixin is known to be an upstream regulator of Rho GTPase signaling at the growth cone. Previous studies have demonstrated that *FRMD7*-regulated neuronal outgrowth may be involved in signal transduction from the plasma membrane receptors to the cytoskeleton [25].

In our study, *FRMD7* was shown to activate GTPase Rac1 signaling in vitro; however, the amount of activated Rac1 induced by the novel missense mutant (c.635T>C) *FRMD7* was obviously decreased. Much evidence indicates that the GTPase Rac1 signaling pathway plays a key role in the regulation of neurite elongation in the developmental stage [26]. Therefore, it can be speculated that its effects at least partly result from the activation of Rac1 signaling induced by *FRMD7*. Mutations of *FRMD7* downregulate the activation of Rac1 signaling, which may be linked to the pathogenesis of idiopathic congenital nystagmus. There are three known regulators of Rac1 GTPase: GTPase-activating proteins, guanine nucleotide exchange factors (GEFs), and the Rho GDP dissociation inhibitor (GDI). Interestingly, FARP1 and FARP2 both function as GEFs, which promote the exchange of GDP for GTP and directly activate Rho GTPases [11,20,23]. However, FERM proteins interact directly with Rho GDI to initiate the activation of Rho small G-proteins. Therefore, the mechanism by which *FRMD7* activates Rac1 signaling, acts as a GEF, or interacts with Rho GDI requires further investigation.

In summary, we have identified a novel missense mutation of FRMD7, c.635T>C, and demonstrated that *FRMD7* activates GTPase Rac1 signaling. However, this signaling is downregulated by this novel mutation, which is therefore implicated in the mechanism underlying the pathogenesis of XL-ICN.
